# The Place of Social Recovery in Mental Health and Related Services

**DOI:** 10.3390/ijerph15061052

**Published:** 2018-05-23

**Authors:** Shulamit Ramon

**Affiliations:** Department of Nursing (Children, Learning Disability and Mental Health) and Social Work, University of Hertfordshire, Hertfordshire AL10 9AB, UK; s.ramon@herts.ac.uk

**Keywords:** the case for social recovery, active citizenship, employment, poverty

## Abstract

This article looks at the place of social recovery in mental health and social care services, alongside personal recovery. Despite its conceptual and practice centrality to the new meaning of recovery, social recovery has remained a relatively neglected dimension. This article attempts to provide an updated critical commentary based on findings from fifty nine studies, including a variety of research methodologies and methods. Definitions of social recovery within the new meaning of recovery are looked at. This is followed by outlining the development and significance of this dimension as reflected in the key areas of shared decision making, co-production and active citizenship, re-entering employment after experiencing mental ill health, being in employment, poverty and coping with poverty, the economic and the scientific cases for social recovery. The article highlights the connections between service users’ experiencing mental health and social care systems, and the implications of ideologies and policies reflecting positions on social recovery. The complexity of social recovery is indicated in each of these areas; the related conceptual and methodological frameworks developed to research this dimension, and key achievements and barriers concerning everyday practice application of social recovery. The summary indicates potential future development perspectives of this dimension.

## 1. Introduction: What Is Social Recovery?

People experiencing mental ill health are referred to in this article as service users or as people, and not as consumers or patients. Most of them cannot afford to buy services and hence cannot be consumers, and all of them are much more than patients within a medicalized system.

Social recovery was defined initially by Warner [1] as economic and residential independence with low social disruption. Today it is defined as people’s ability to lead meaningful and contributing lives as active citizens while experiencing mental ill health [2].

Social recovery is perceived here as a key dimension of mental health recovery, albeit a relatively neglected one within the conceptual framework of the new meaning of recovery, as well as in recovery practice and research [3]. This neglect is due to the placement of mental health recovery as primarily a clinical and medical issue. Following Bourdieu [4], social recovery is perceived to be the journey of people experiencing mental ill health towards regaining social recognition and acceptance, in the form of their social identity and presence. 

As distinct from personal recovery, yet inter-related to it, social recovery includes the components of interdependence with others, connectedness [5], recovery capital [2] and social capital [6], as well as the impact of collective culture and the structural elements of our socio-economic-political system. To add to the complexity, the impact of each element on one’s identity, in interaction with how one is seen by others, needs to be taken into account.

Reference to social recovery is usually made when referring to social inclusion, exclusion, or stigma. While these are important dimensions of social recovery, some of which are looked at in other articles of this special issue, the exclusion of the dimensions outlined above leads to a too narrow understanding of the concept and its significance. 

Given the interdependency mentioned above, only an analytical separation exists between the personal and the social dimensions of recovery in mental health. Both co-exist in the life trajectory of a person and that of their social group.

Personal recovery has been defined as ‘a deeply personal, unique process of changing one’s attitudes, values, feelings, goals, skills and/or roles, in a way of living a satisfying, hopeful and contributing life even with the limitations caused by illness’ [7], p. 16. There is only one element in this definition which hints of the existence of a social dimension, namely that of “contributing life”, though it is not specified what this contribution is likely to mean. This definition does not use explicitly the concept of social recovery, and appears to assume that having a meaningful life, ability to live well with one’s symptoms may take place without an explicit social dimension.

An alternative definition is provided by Davidson [8]. It focuses on living with the illness and living beyond the illness. Living beyond the illness allows the introduction of an additional dimension in the lives of people with the lived experience of mental ill health, where they can not only be in the community, but be also its active members [9]. 

When this group of people are asked what has made a positive, qualitative, difference which enables them to move into the journey of recovery, the intuitive response they give is having people who believe in them [10] ([11], pp. 9,11). This response encapsulates not only the interdependence we have with others, but also the relational aspect, the connectedness, the solidarity, and the importance of our social belonging.

The following key areas of social recovery will be explored below:

- shared decision making, co-production and active citizenship
- employment
- living in poverty
- the economic case of recovery
- the scientific evidence for the recovery model

Relevant insight into aspects of social recovery is also provided in this special issue in Byrne’s paper on peer support workers, Henderson’s paper on stigma, and Latimer’s paper on housing.

## 2. Note on Methodology

This paper takes the form of a qualitative commentary on social recovery, based primarily on findings from relevant research published in English between 2000 and 2018. 

Fifty nine papers and books were looked at. They included systematic reviews (ten), reviews (ten), applied mixed qualitative and quantitative methods (twelve), applied only qualitative methods (fifteen), applied only quantitative methods (five), had a Randomized Control Design (three), were longitudinal studies (two), were experiential in nature (two).

Given the subjective nature of any type of recovery, and the relative paucity of publications reflecting service users and carers’ experiences, it is important to include reflections of recovery journeys alongside formal research. Countries in which the studies were conducted included Canada, Israel, Spain, Sweden, UK and the US.

## 3. Shared Decision Making, Co-Production and Active Citizenship

### 3.1. Shared Decision Making

Shared Decision Making (SDM) in mental health focuses on the process of making decisions related to mental health interventions jointly, either by a dyad consisting of the person and their service provider, or by a triad which also includes the person’s carer, and/or by a relevant network of which the person is a member [12,13].

Developed first in the context of terminal physical illness [14], the application to mental health continues to be developed. The assumptions underlying SDM include the equal importance of sharing experiential knowledge and scientific knowledge between two or more experts (the person experiencing mental ill health as an expert alongside the service provider, who is an expert too), each of whom brings knowledge the other does not have to the process. Key elements of the process include understanding the problems faced currently, knowledge of the preferences the person has, the positive and negative impact of previous interventions, understanding what the proposed interventions are likely to offer and what their adverse effects may be, and attempting to reach an agreement concerning future action. Although formally such a process should have taken place in mental health systems, we know from the evidence of many service users of mental health services that this is usually not the case [15]. All too often the full information about interventions is not given, or not given in a way that can be understood by the person, the provider presents their suggestions as the best available, and is not interested in the person’s views and preferences. Being treated as a person, rather than as a bundle of symptoms and problems, is presented by service users as the key condition to being ready to share with the provider their genuine thoughts and preferences [16,17,18]. Knowing that the provider has the power to lead to a compulsory admission, or/and use of constraints on a ward is a major factor in preventing people from sharing with the provider their genuine preferences [19]. Hence making decisions about oneself and thus taking responsibility for oneself are often blocked.

Existing research demonstrates that most people experiencing mental illness are able to make decisions and have the mental capacity to do so most of the time, including many of those who are in an acute admission ward [20]. This is hardly surprising to those of us coming from the recovery perspective, given that the intellectual and social capacities of many members of this group have been demonstrated by the strengths approach [21], the valued contribution of peer support workers and the impressive contribution of service users who have championed recovery [22].

The process of SDM is relatively simple and needs little technological support. Although it takes more time initially to get used to this shared way of reaching decisions, the time needed once it becomes part of everyday routine, is reduced. It is possible to provide training to all involved groups in a co-produced way by both service users and professionals, as well as adults and young people [23], but this very necessary option is hardly applied at present. Co-produced training is possible once the value of such an involvement is accepted by professionals, service users and carers, together with the importance of the contribution made to SDM by service users as co-leads.

The dyadic relationships in which SDM is often practiced represents power relationships far beyond the two people engaged in the SDM process. The service provider represents society, and s/he is mandated to ensure that specific social norms are adhered to, within the care and control range allocated to mental ill health in a specific society. The service user represents those who have deviated from the norms, but did so due in part to their social conditions, as well as a reaction to adverse life experiences, the outcomes of which they could not control. The new meaning of recovery added the belief in their strengths, and in their wish to become active citizens, as tools that enable them to do so with social support.

While most of the existing action research projects on SDM have focused on medication decisions, projects looking at the wide range of social recovery decisions—such as family relations, education, employment, housing and leisure activities are beginning to emerge [24,25]. Given that the scope of such activities is much wider in the context of the recovery journey than that of medication, it is central that SDM will be practiced within this area too.

Shared decision making in mental health is hardly implemented in everyday practice [26], even though it has the potential to support co-production and active citizenship of service users, and thus contribute to changing the existing power differential between service users and providers, as well as the social place of this group of people.

### 3.2. Co-Production in Mental Health

Co-production as an important dimension of recovery has begun towards the end of the 20th century, but has developed further in the 21st century [27]. It is based on the belief that service users have not only strengths to share in a joint project, but that co-production can enhance the power they have within such an undertaking, and with it their social standing and identity, as well as enriching any given project. Participation as equals in the management of projects is a good example of co-production (see an example in The Haven initiative, [28]; or in co-training and co-researching in mental health shared decision making of young people and adults [23,25]. The power parity is evident in these examples, as well as the value given to the contributions made by the different participants.

Roper, Grey and Cadogan [29] look at the principles, problematic aspects, and necessary conditions for the success of co-produced projects in mental health, providing a number of useful examples from Australia and the UK. They perceive co-production to be based on social justice and community development, even though it comes originally from economics [30]. For them, the essence of such an approach in mental health is reversing the existing power imbalance by enhancing the power and leadership of service users to enable them to share their unique knowledge and experiential expertise. The necessary conditions for the success of co-production include giving support and time for service users to become better able to act as leaders and knowledge providers, focus on the process of co-production, and for all participants to be open about differences and potential conflicts, rather than to sweep these under the carpet. A high degree of openness is asked not only from service providers. In the example given service users who are trainers and object to taking medication need to be ready to understand the perspective of those who wish to take medication and accept it as their legitimate choice [29], p. 23.

The recognition that co-production requires a lot more time to complete a project than a non-co-produced one is a reflection of the need for a process in which suitable modes of communication are found to fit with the emerging power, leadership and contribution to the project by the service users. Likewise, a process of readjustment is necessary also for the non-service users, who may be professionals or representing established organizations [30].

Examples of co-production projects in practice include developing a tool for dialogue on ECT between service users and providers to support the beginning of a consultative process, in which the service users were encouraged to ask all of the questions they had, make them comfortable when asking, and encourage more informed choices to be taken up by them [29], p. 19.

Critical elements for co-production include having everyone on board, support for the initiative before it begins, willing to take risks, and access to co-production expertise and support if needed.

Participatory action research (PAR) is another way of enabling co-production [31]. It enables the acquisition of new skills and interests, such as learning what is research, which are the relevant methods for a PAR design, how to do it, how to contribute to data analysis, write up and presentation of research findings. Beyond the development of new skills come the benefits for one’s social status and identity, and the potential opening of new opportunities for training in research and for participating in other research projects. An interesting example is provided by Mahone and her colleagues of a PAR study between a public mental health clinic and a university nursing department [32].

Co-production has the potential to generate a higher degree of social inclusion, but also new knowledge and evidence.

For a number of service user groups co-production is perceived to be insufficient in redressing the power imbalance, and they aim at separate organizations which include only service users [33]. The attraction of such an arrangement is easy to understand in terms of being finally in full control and having the power to decide on their own with whom to share their activities. Developed by service users initially, the new meaning of recovery has been an empowering concept in particular for this group. However, the focus on the uniqueness of any one group may prevent the full use of the expertise which exists beyond one group, hence preventing opportunities for genuine co-production.

In summary, co-production is neither easy nor simple to achieve, given a history of institutionalization, prevailing perception of mental illness as only a bio-medical issue, lack of joint work history, and a belief that having a mental illness renders people unable to have a meaningful contribution to offer, which all too often becomes also an internalized identity.

Putting in place processes of shared decision making in mental health, in dyads or triads (person and clinician, person, clinician and carers) [23,26,34] and in networks (e.g., the Open dialogue, [13,34]) is a necessary, yet insufficient, condition for co-production, as an enabler for the re-discovery of hope, abilities, and rejoining ordinary living.

### 3.3. Active Citizenship

The concept of active citizenship in the context of social recovery in mental health relates to the wider discussion of biological citizenship [35], medicalization and de-medicalization, as well as to the social model of disability [36]. Given the contested nature of mental ill health and its likely underlying causes, this issue is often fudged by formally applying to it bio-psycho-social lenses. However, in our social reality which is dominated by the belief in science and medicine, both the psychological and the social aspects of mental ill health receive less attention than the assumed bio-medical base. The biological citizen, a concept focused on being governed and of taking an active part in self-governing, encourages up to a point the individual aspect of self-governing. The latter is accentuated within certain social ideologies, such as neoliberalism, to the point of blaming individuals for failing to succeed economically and otherwise, by ignoring the impact of social structural factors, and acting as a justification for the destruction of protective frameworks, and often demonizing the citizen who is perceived as a failure [37,38].

The social model of disability [36], which is of relevance to the new meaning of recovery and to social recovery, provides an example of citizens self-governing as against being governed by society. It highlights the role of society in the stigmatization of people with disabilities and in erecting a number of barriers they face in leading an ordinary life. It does not accept that disabilities inevitably limit people’s abilities, and some of its protagonists call for the affirmation of a disabled identity as superior to that of non-disabled people [39].

The new meaning of recovery straddles a complex path in following mainly a non-medicalized approach, without denying the place of medication within the range of mental health intervention. It seems to accept without discussion the multiplicity of underlying causes leading to mental ill health, though the writings of recovery oriented thinkers often favor psychological and social factors, such as trauma, abuse, stigmatization and social deprivation over biological factors [40]. It also focuses on care and not on cure, and emphasizes leading a meaningful life with the illness and beyond it. A meaningful life beyond the illness includes fostering personal and collective responsibility towards oneself and others. The strong belief in the potential and actual strengths of people with the lived experience of mental ill health, and the empirical evidence supporting this belief [21,22] provide the basis for the assumed abilities this group has with which to achieve such responsibility. The social recovery dimension emphasizes the place of social structural factors as impacting on the range of individual and collective choices available to people in their recovery journey, which in turn impact on their self-governing capacity. By advocating active citizenship as a recovery-oriented objective, social recovery widens the scope of individual and collective governing.

Citizenship in the context of mental health is defined as “the strength of a person’s connections to the rights, responsibilities, roles, risks, resources and relationships that society offers to people through public and social institutions” ([41], p. 1). Developed particularly by Rowe at Yale’s recovery program, but since developed also in Canada, Scotland and Spain [41], active citizenship is an attempt to enable people with the lived experience of mental ill health and related difficulties (such as homelessness) to explore the options in which they can contribute to the wider community, and advocate for change in mental health systems. This can take many forms, such as beginning by membership in a mutual support group, moving to represent that group in a larger forum, and/or being active in their local community, on a range from a local family circle to membership in a political party. The value of such activities lies in enlarging one’s meaningful network, moving from being a passive to an active citizen, being validated by other people in the community, learning skills necessary for the specific activity, learning more about one’s potential and one’s strengths, and becoming motivated for further such activities due to the success experienced. The fact that many such activities take place outside the arena of mental health services is a bonus, as it expands and reinforces people’s connectedness, living beyond the illness, and their recovery capital.

Empirical research has demonstrated that those service users who develop their citizenship activities increase also their level of recovery [9]. This inter-relationships is not surprising, because being an active citizen enhances one’s personal and social identity, as well as enabling the acquisition of new skills and enriching one’s network [6]. At present, good correlations between these two variables are noted, rather than a clear causal relationships. Pelletier et al. [9] have carried out an exploratory factor analysis and a confirmatory factor analysis between a measurement of citizenship (CM) and a measurement of recovery (RAS) which has highlighted the centrality of personal confidence, hope, willingness to ask for help, goal and success orientation, reliance on others, without domination by symptoms ([9], pp. 5, 6).

Becoming an active citizen is not a dimension that mental health service providers can do for people using the service; the latter need to do this themselves. Yet there is a range of opportunities and networks which facilitate this achievement which providers can support. This process is exemplified in the Barcelona development of active citizenship there, where a variety of local and EU mental health initiatives have led to creating a sound base for this development [41].

Rowe et al. [42] describe how the focus on active citizenship has also led him and his colleagues to develop co-production with the participants in the form of participatory action research (PAR), already mentioned in the section on co-production (see p. 4). Both shared decision making and co-production are enablers of active citizenship.

## 4. Employment

In the context of mental health there are two key facets related to employment:

(1) Employment for people experiencing mental ill health
(2) Responding to mental health difficulties experienced by people in employment

### 4.1. Employment for People Experiencing Mental Ill Health

Being in employment is perceived world wide as a socially desirable position, in terms of a marker of a socially inclusive identity, social status, income, opportunities for networking and skills acquisition, being a contributor to the production and to the wealth of one’s country. As such it fits well the aims of the recovery journey, and forms part of the person’s social recovery. Existing evidence highlights that once in work the diagnosis attributed to the person does not matter in terms of predicting likelihood to stay at work [1] and that given the right support employees with mental ill health tend to be more devoted to the workplace than those without this experience [43].

Availability of work for this group is higher when economic market success is higher, than when it is lower [1]. The rate of employment among people with the lived experience of mental ill health is poor in most countries [43]. In the UK 32–34% of the people identified as having a mental illness are in full employment, while 66% of women in this group are unemployed or economically inactive, compared to 56% of men [44], p. 59. However, according to the TUC report [45] that only one in four people with long term mental illness is at work (2017 figures: only 26% of those experiencing severe mental ill health, only 45.5% of those experiencing depression or anxiety).

Research carried out by Tilburg University for IOSH [46] in which both professionals working in health safety as well as employees experiencing mental ill health took part, highlighted that barriers to returning to work included excess of workload, but not one’s mental health condition. Facilitators included gaining self-awareness and setting limits, having a supportive manager, positively valuing one’s work, regaining control by engaging in recovery enhancing behavior.

People who do not work need to explain to themselves and to others the reasons for this state, and are often perceived as unproductive members of society. This attitude is prevalent in all countries which have embraced the neoliberal ideology, clearly expressed in treating unemployed people with a disability as a burden to society, and hence as a socially undeserving group. This approach is reflected in the yearly decrease in the real value of disability benefits, and in the largely controlling way this group is treated by those responsible for administering the benefits to them.

People have to demonstrate that they are actively seeking employment, and their benefits may be cut if their behavior does not satisfy government’s officials. It takes a long time to re-instate the payment of benefits, and in the meantime people—and their dependents—may be literally starving.

Moreover, having a reasonable level of financial benefits is described by highly reputable mental health researchers as a “benefit trap”, which allegedly prevents people from seeking employment [47]. This seems to be an additional layer of the stigma attached to people who experience mental ill health, instead of changing the inflexibility of the existing state system to enable people a gradual move to employment without losing their right to housing, or to other benefits, if they work more than the specified number of hours per week.

Housing is the most costly element of the financial benefits available to people with a high level of disability, including mental ill health [48]. Most jobs, even if full time, do not provide an income level that would cover the cost of housing. Thus people are faced with the dilemma of losing their housing benefits with no alternative housing solution or staying on benefits. This problem cannot be resolved by individuals who use mental health services, but by governments and local authorities. Hence to blame these individuals is not only unjust, but is adding to the stigma they already live with.

IPS (Individual placement and support) is a scheme focusing on supporting people to enter competitive employment by providing individualized long term support in the workplace [46] for people with all types of diagnoses. Originating in the US, it has proven to be the more successful way of becoming and staying employed [49], not only in the US, but also in Europe [50].

The outcomes of people entering competitive employment and staying there, while in parallel not being in need of frequent hospitalization episodes, are accounted for by the individualized nature of the scheme, its main location within the employment base, and by the readiness of the employer and other employees to foster it.

However, introducing IPS to South London and other parts of the UK has not been a success story [51] for a variety of reasons. Schneider [52] explains and exemplifies the key reasons which are mainly located at the inter-organizational meeting point. To be a success, IPS requires co-location and joint work of IPS training team with the clinical team throughout the duration of the scheme. Such joint work is not easy to achieve even if the teams are physically located in the same building, because of differences in status and perspectives on mental health and the capacity of people with long term mental ill heath to work successfully in a competitive environment. Changing professional mentalities usually requires a lengthy process and competent, dedicated, leadership, one that does not match the logic of a time limited project. The lack of attempt to have co-leadership of service users has not been looked at in any of the IPS projects.

### 4.2. Responding to Mental Health Difficulties Experienced by People in Employment

The rate of mental health difficulties experienced by people in employment is estimated at 60% of the total, with only 30% known to be service users of mental health services [53].

Most of the difficulties relate to anxiety and depression, which are defined as common mental health diagnoses. It is unclear as to what extent the reasons for these difficulties relate to work conditions and/or personal relationships issues, but it makes sense to assume that both factors are at play [54]. Workers in the health sector fare worse than those in other sectors in having a higher rate of mental ill health [55].

The factors leading to experiencing these difficulties include long working hours, being overloaded, work pressure, lack of control, lack of participation, poor social support, unclear management and work role, and the overall impact of the above on employees’ personal lives [56]. It is also relevant to remember that the impact of an individual experiencing mental ill health spreads often also to the teams they work with in a variety of ways.

The level of such difficulties has been sufficient to cause concern for employers, trade unions, and mental health professionals, especially given the high financial cost of employees being unwell, unable to perform to their full capacity, and at times absent from work. According to the Centre for Mental Health [55] the financial cost to the UK is estimated at £34.9 billion, comprising of staff turnover (£3.1 b), reduced productivity (£21.2 b) and sickness absence (£10.6 b).

In the context of social recovery key issues here are the likelihood of preventing the situation from escalating to the point where the person cannot continue to work, the usefulness of current responses to the high level of this problem, and the treatment of underlying contributing work conditions.

Two systematic reviews of this issue [53,56] perceive the workplace problems to be amenable to change in most work situations, bar redundancies. Interventions vary from stress management, role play, improving communication, improving empathy, to organizational level interventions. The latter include methods of enhancing control of employees mainly through committees, and focus on problem solving [55].

The demand-control-Support (DCS) model, tackled by participatory intervention, seem to lead on the whole to positive outcomes in terms of reduction of stress and absenteeism, but not when exercised in redundancy cases [56]. Although accepted by social policy makers in many countries as necessary, and although most large work places have either their own mental health services or contract to outside services, few of them have increased employees’ participation in the workplace neither before the economic crisis of 2007–2008, nor since.

## 5. Poverty

### 5.1. Living in Poverty

Poverty is defined as living below the average income of a given society, and thus has an in-built relative dimension. It is usually the outcomes of an unequal distribution of wealth in a given society, where preference is given to the principle that individual earnings dictate this distribution.

The majority of people experiencing mental ill health which disables them from working live in poverty; experiencing mental ill health may bring people to experience poverty [57].

Poverty is not limited to low income, as the latter imposes constraints on what people can afford in terms of nutrition, clothing, housing, education, travelling, participation in cultural and leisure activities. It also implies a shorter life expectancy of the people who live in poverty compared to people living in the same neighborhood who are better off, or huge differences in longevity rates between one end of a country and another end of it [58].

If receiving financial benefits, they have to prove their eligibility for them, and are labelled as “unproductive” and a “burden on society”. Thus poverty brings often social exclusion, stigma and discrimination with it. According to Mills [59] “when people living in poverty are asked about the lived realities of their experience, it seems that some talk about despair, pain, being driven mad, and losing the desire to live” (p. 213).

Furedi [60] states that society feels more comfortable dealing with poverty as a mental health problem, rather than as a social structural issue. This is the case partly because the mental health label enables individualization and illness attribution, which reduces individual and social responsibility to different extent in different countries, depending on their attitudes to people with disabilities, expressed in their welfare policies.

The connections between increased poverty and mental ill health have been demonstrated in a number of ways. Barr, Taylor and Stuckler [37] conducted a longitudinal study in 149 English local authorities on the relationships between poverty and mental health between 2004 and 2013. Their findings highlight that in former industrial heartlands there was a high rate of suicide (double the national average), 725,000 added prescriptions for antidepressants, and 279,000 new cases of self- reported mental health problems. These areas had 5% of all national suicides, 5% of total antidepressants prescription, and 11% of self-reported mental health problems.

UK suicide rates show that fit-to-work-assessment is linked to increase in suicide figures among people with disability, which have gone from 21% to 43% in the last seven years [38]. These assessments, instructed by the Department of Work and Pension, can determine if people are not eligible to continue to receive unemployment or disability benefits.

Before the economic crisis of 2007–2008 Greece had the lowest rate of suicide in Europe. By 2012 the rate has doubled (http://thebodyeconomic.com/ accessed on 27 March 2018).

According to Elliot [57] the combination of poverty and mental ill health leads to “a corrosive impact of stigma and discrimination on people experiencing mental health problems and those living in poverty” (p. 4). People experiencing long term mental illness have a much lower life expectancy than the rest of the population, estimated at 20 years less, partly explained by the impact of living in poverty, lack of attention to their physical health [58,61] and the impact of the polymorphous medication they are given [62].

The population rate of mental ill health goes up in lower socio-economic neighborhoods in the UK to 26% for women and 23% for men in high risk, with 75% of them not receiving ongoing treatment ([57], p. 7).

### 5.2. Coping with Living in Poverty

Topor and his colleagues working in Stockholm have carried a series of qualitative studies on how people with lived experience of mental illness and poverty manage their lives. Their analysis highlights the impact of what they call “double trouble” [63] of the mixture of severe mental ill health and poverty on people’s lives, in terms of increasing their social isolation, diminishing their sense of agency and mastery, ability to reciprocate and maintain the social network they had.

The experiment of giving people an additional small sum of money ($73) on top of their usual income and letting them decide what to do with it, described in their recent paper [64], illustrate how easy it is to for them to switch to the use of money with agency and mastery, to feel good about being able to entertain friends and hence to become less socially excluded.

The paper on friendship [65] focuses on what it means to manage life on the margin, using strategies that are similar to those used by people living in poverty without mental ill health. These strategies include staying within limits, widening the boundaries and indulging in the unnecessary.

Relying on relatives, in particular parents, for financial support is also a typical strategy available to those lucky to have caring relatives.

Benbow et al. [66] propose the application of the capability approach, developed by Sen and Nusbaum [67] and further by Wallcraft et al [68] to understanding poverty and social exclusion of psychiatric survivors. As part of a longitudinal study, they investigated the views of 380 people who experienced homelessness, mental ill health, and being treated by the social justice system in Canada as to how it felt to be in such a social place. Poverty, stigma, belonging, and the right to be advocated for were the key issues the sample came up with. Rudnik et al. [69] (who participated in Benbow’s study) concentrate on the wishes of people treated by the social justice system, through the findings of a focus group with 34 people, applying an ethnographic approach. The participants wanted support in self-determination, much more in-depth focus on meeting basic needs and for a good quality service system.

All of these papers indicate that a socially contextualized approach is necessary in the attempt to understand and work with people experiencing the duality of poverty and mental illness. Once such an approach is applied, psychiatric symptoms can be understood as rational coping strategies in the reality of poverty.

## 6. The Economic Case for Mental Health Recovery

Given the high level of spending on mental health services in the Western world the cost effectiveness of such spending becomes an important issue. Cost effectiveness includes usually not only the economic cost, but also the added value of different dimensions to people’s quality of life due to an intervention, and focuses on the overall effectiveness in terms of outcomes [70].

Two recent publications look at the economic case of recovery [71,72] in somewhat different ways. While Knapp et al. look at outcome studies in term of their recovery effectiveness, Slade et al. look at research evidence, service users and carers’ narratives and economic evidence for ten challenges that they see as the key to effective recovery ([72], p. 4, box 2). While both publications look at the case for crisis focused services, employment, housing, peer support work, personal budgets, and recovery colleges, the other dimensions differ. Knapp et al. [71] look at self- management, economic safety net (welfare and debt advice), education, housing, physical health promotion, personal budgets and stigma campaigns. The challenges focused on in the Slade et al. publication include organizational change, leadership, risk assessment and management, workforce attitudinal and practice change, and staff support.

The section on the economic evidence concerning safety is of particular interest due to the shift in perceiving risk in mental health as something to be avoided, to an issue worthy of co-production to improve safety and quality of life. The economic evidence highlights the cost of the use of restraints to the restrained person and to staff working with them. For example, the cost of a violent incidence leads to an increase of 54% in staff sickness, while the increase in agitation and distress to service users in the wake of applying physical restraints leads to increase in the cost of care to three times more than that of a psychological intervention [73]. Existing evidence highlights that the systematic engagement of service users as trainers reduces dramatically the use of constraints [74].

Perhaps because Slade et al. give more space to research studies and to narratives, as well as to economic evidence, they are able to provide a more nuanced approach which tells us what of the different dimensions of recovery carry more evidence with them.

In both publications by Knapp et al and Slade et al, dimensions related clearly to social recovery—such as education, employment, dispersed housing, economic safety net, and reducing risk without using constraining measures—carry more available economic evidence than dimensions related to strictly personal recovery.

## 7. The Scientific Case of Social Recovery

Richard Warner’s succinct summary of the scientific evidence related to the recovery approach [75] is primarily focusing on social recovery. He highlights the achievement of this approach in looking at four areas:

(1) The success of the many people who left long term institutions to lead a reasonable life in the community in terms of economic and residential independence with low social disruption, even if their symptoms have not disappeared.
(2) More people experiencing mental ill health have become empowered
(3) Shared decision making is developing
(4) Productive roles have been found for this group.

## 8. Conclusions

It is hoped that the case for recognizing the centrality of social recovery as an essential part of the new meaning of mental health recovery has been made successfully in this paper, as well as highlighting the need to look at it consistently alongside personal recovery.

The relative neglect of this component in research is perhaps related to its multidimensional construction, the dominance of the bio-chemical approach to mental ill health, and the difficulty in changing social structural elements. While lending itself to both qualitative and quantitative research methods, the multidimensionality does not lend itself easily to randomized control trials.

The paucity of policy making, and of appropriate budgeting concerning social recovery, are related to the dominance of neoliberalism in most of the societies which accept in principle the value of the recovery approach in the field of mental health. The demonization of those living in poverty in most capitalist societies effects also those who experience mental ill health.

The lack of the much necessary community work in mental health and social care services is also a function of the impact of neoliberal ideology, while constituting a key barrier to attempting to foster social recovery in practice. Greater awareness of the conceptual aspect of social recovery has taken place since 2000, in particular in relation to the concepts of social capital and recovery capital, which complement the strengths approach.

The emerging focus on co-production and active citizenship to overcome social exclusion and to foster social inclusion of people experiencing mental ill health is encouraging, and hopefully will also enhance the implementation of shared decision making at all levels. The message of social recovery lies in the need to include the social context in understanding, analyzing, and responding to people’s mental health difficulties. While Knapp et al. [68] and Slade et al. [69] have outlined the economic case of mental health recovery, Warner [72] has outlined convincingly the scientific evidence of social recovery.

This author, for one, shares Warner’s optimism while being aware of the obstacles to achieving social recovery for all who need it.
